# Organ-specific adaptive signaling pathway activation in metastatic breast cancer cells

**DOI:** 10.18632/oncotarget.3707

**Published:** 2015-03-30

**Authors:** Riesa M. Burnett, Kelly E. Craven, Purna Krishnamurthy, Chirayu P. Goswami, Sunil Badve, Peter Crooks, William P. Mathews, Poornima Bhat-Nakshatri, Harikrishna Nakshatri

**Affiliations:** ^1^ Department of Surgery, Indiana University School of Medicine, Indianapolis, IN, USA; ^2^ Department of Biochemistry and Molecular Biology, Indiana University School of Medicine, Indianapolis, IN, USA; ^3^ Department of Pathology and Laboratory Medicine, Indiana University School of Medicine, Indianapolis, IN, USA; ^4^ Department of Center for Computational Biology and Bioinformatics, Indiana University School of Medicine, Indianapolis, IN, USA; ^5^ University of Arkansas, Little Rock, AR, USA; ^6^ Leuchemix, Inc., Woodside, CA, USA

**Keywords:** breast cancer, brain metastasis, NF-kB, DMAPT, TMEM47

## Abstract

Breast cancer metastasizes to bone, visceral organs, and/or brain depending on the subtype, which may involve activation of a host organ-specific signaling network in metastatic cells. To test this possibility, we determined gene expression patterns in MDA-MB-231 cells and its mammary fat pad tumor (TMD-231), lung-metastasis (LMD-231), bone-metastasis (BMD-231), adrenal-metastasis (ADMD-231) and brain-metastasis (231-BR) variants. When gene expression between metastases was compared, 231-BR cells showed the highest gene expression difference followed by ADMD-231, LMD-231, and BMD-231 cells. Neuronal transmembrane proteins SLITRK2, TMEM47, and LYPD1 were specifically overexpressed in 231-BR cells. Pathway-analyses revealed activation of signaling networks that would enable cancer cells to adapt to organs of metastasis such as drug detoxification/oxidative stress response/semaphorin neuronal pathway in 231-BR, Notch/orphan nuclear receptor signals involved in steroidogenesis in ADMD-231, acute phase response in LMD-231, and cytokine/hematopoietic stem cell signaling in BMD-231 cells. Only NF-κB signaling pathway activation was common to all except BMD-231 cells. We confirmed NF-κB activation in 231-BR and in a brain metastatic variant of 4T1 cells (4T1-BR). Dimethylaminoparthenolide inhibited NF-κB activity, LYPD1 expression, and proliferation of 231-BR and 4T1-BR cells. Thus, transcriptome change enabling adaptation to host organs is likely one of the mechanisms associated with organ-specific metastasis and could potentially be targeted therapeutically.

## INTRODUCTION

Breast cancer brain metastasis is a growing public health concern as advances in systemic therapy have helped to contain metastatic growth in most organs except the brain [1]. Brain metastasis occurs in 10-15% of patients with metastatic breast cancer [2-4], and is associated with an extremely poor prognosis with a median survival of only 3-6 months [5, 6]. Patients with HER2+ or triple negative breast cancer (TNBC) have a greater propensity to develop brain metastasis [7-13].

Three processes may control brain metastasis. The first may involve a minority of primary tumor cells with unique mutations that impart proclivity for brain metastasis. Recent massively parallel sequencing of primary tumor and a brain metastasis from the same patient suggested this possibility [14]. The second is that mutations and/or epigenetic changes in cancer cells bestow blood-brain-barrier (BBB) permeability and consequently brain metastasis. The third is that every cancer cell has the ability to reach the brain but only a few cells that can acquire neuronal cell function through either additional mutations in cancer cells or brain microenvironment-induced epigenetic changes in cancer cells that are essential for metastatic growth proliferate in the brain. For example, circulating tumor cells that metastasize to the brain overexpress proteins such as heparanase (HPSE) that allow cancer cells to interact with brain vasculature [15]. Brain metastatic cancer cells express SERPINE1, which helps in vascular co-adaptation in the brain [16]. The Biology of Brain Metastasis Workshop organized by the National Cancer Institute (NCI) has set several research priorities with respect to biology of brain metastasis [17]. These include investigations into the pathogenic mechanisms of metastasis to brain, identification of commonalities and uniqueness of brain metastasis versus other sites of metastasis, differentiation of indolent and aggressive lesions by understanding heterogeneity among different brain metastatic lesions, investigation of the relationship between “stem cell” features and brain metastasis, and understanding the mechanisms responsible for tumor cell homing to the brain.

Progress in addressing the above issues is limited largely due to the lack of suitable model system. Most of our current knowledge on brain metastasis is derived from studies using brain-seeking variants developed from HER2 -amplified BT474 cells and triple negative breast cancer/mesenchymal stem cell-like cell line MDA-MB-231 [18, 19]. Analyses of MDA-MB-231 derivatives enabled development of a brain metastasis signature and identification of a set of genes that may be involved in BBB extravasation. Genes identified in these studies include the brain-specific sialyltransferase ST6GALNAC5, COX2, ANGPTL4 and EGFR ligands epiregulin and HBEGF [20]. NF-κB inducible genes MMP-1 and FSCIN-1 are also associated with brain metastasis [21]. In experimental models, brain-seeking metastatic variants but not the variants that metastasize to other organs have the ability to establish a unique pattern of vascularization required for growth [22]. However, gene expression changes in brain metastatic cells as an adaptive response in the brain microenvironment are just beginning to get attention.

To begin to address these complexities, we compared gene expression patterns in cancer cells isolated from a brain metastasis with parental cells in culture, mammary fat pad tumor-derived cells, and cancer cells that have metastasized to lungs, bone, and the adrenal gland. We identified a set of genes that are upregulated only in brain metastatic cells compared with all other cell types. Several of these genes have neuronal function suggesting that these genes are “reactivated” in the metastatic cell to enable them to adapt to growth conditions in the brain and utilize neuronal signaling networks for their advantage. Comparison among cells isolated from different metastatic sites revealed significantly higher transcriptome changes in brain metastatic cancer cells and unique pathway alterations involved in drug detoxification. In general, metastasis, irrespective of organs of metastasis, was associated with gain of gene expression suggesting that hyper-activation of general transcriptional machinery is a contributing factor of metastasis.

## RESULTS

### Brain metastatic variants of MDA-MB-231 (231-BR) cells expressed a unique set of genes compared with parental cells, mammary fat pad tumor, or variants from other organs of metastasis

We recently reported an organ-specific metastasis model of MDA-MB-231 cells that included establishing cell lines from metastases in the lung, the bone, and the adrenal gland [23]. The same cell line model has been used to develop brain metastasis variants [24]. Using these cell lines, we had demonstrated upregulation of 20 and downregulation of seven microRNAs in metastatic cancer cells compared with mammary fat pad tumor cells [23]. We subjected parental MDA-MB-231 cells from two labs (one from us used for developing tumor and metastatic variants except brain metastasis and the other used for developing brain metastatic cells- these cells are labeled MD-231P), mammary fat pad tumor derived cell line (TMD-231), lung metastasis (LMD-231), bone metastasis (BMD-231), adrenal metastasis (ADMD-231), and brain metastasis (231-BR) to microarray mRNA expression analysis. For the different sets of cell lines, we used PAM [25] to identify signature genes for a specific metastasis site compared with all other sites. PAM classifier is based on the nearest shrunken centroid algorithm and identifies signature genes based on the variability of genes in a group. Using this method, we compared each metastatic site's gene expression profile to all other metastatic expression profiles, tumor-derived cells, and parental cells to compile a set of genes constituting a signature for that metastatic site only. This stringent analysis generated signatures that were unique to brain and adrenal metastasis (Table S1). However, lung and bone metastasis signatures were not as statistically robust as brain and adrenal signatures and demonstrated a higher error rate (Table S1).

**Figure 1:**
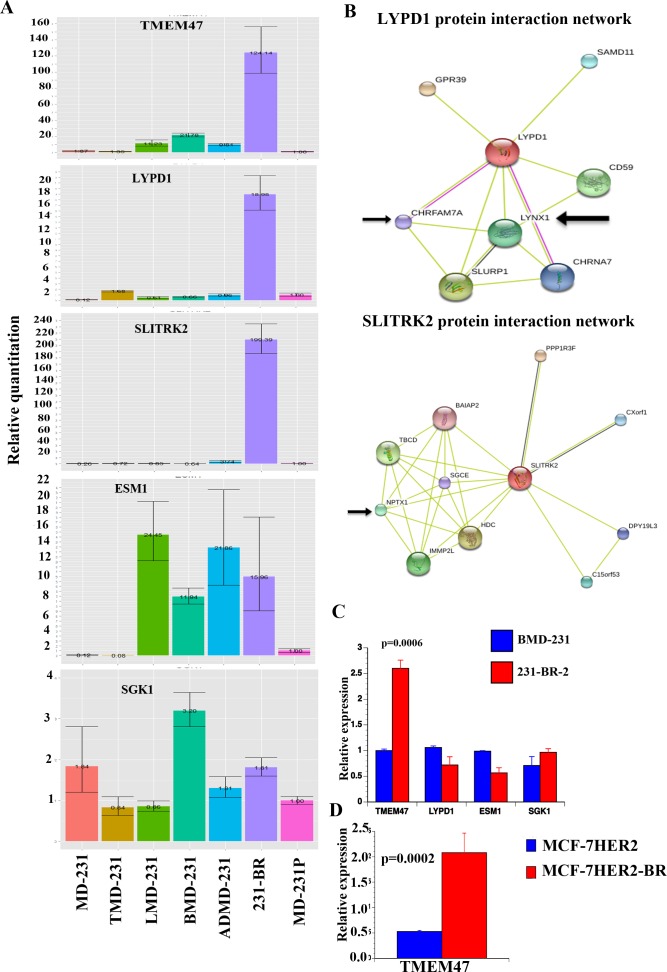
Validation of genes differentially expressed in brain metastatic cells **A**) qRT-PCR analysis of select genes in parental, tumor-derived, and organ-specific metastatic cells. β-actin was used as a normalization control. **B**) Protein-protein interaction network of two genes expressed preferentially in 231-BR cells. Data were generated using STRING network [31]. Arrow indicates proteins involved in neuronal signaling. **C**) TMEM47 expression is elevated in another brain-metastasis variant of MDA-MB-231 cells. This variant was derived from BMD-231 cells. **D**) TMEM47 expression in MCF-7HER2 and its brain metastatic variant.

231-BR cells showed upregulation of 396 genes and downregulation of 77 genes compared with all other cell types (*p* < 0.01) (Table S2). In general, metastatic cells showed a higher number of upregulated genes compared with MDA-MB-231 or TMD-231 cells suggesting that acquiring new gene expression rather than loss of gene expression is associated with metastasis (Table S2, see rows 1 and 2). The top 25-upregulated genes in 231-BR cells are shown in Table 1. Several genes in this table (indicated in bold) are linked to neuronal activity. For example, translation elongation factor eEF1A2 variant is expressed in a restricted pattern compared with ubiquitously expressed eEF1A1, and the expression is dominant in adult brain [26]. TMEM47 and SLITRK2 are linked to neuronal development and/or brain tumors [27, 28]. TMEM47 is also called brain cell membrane protein 1 and is related to claudins [29]. Thus, upregulation of genes linked to neuronal function in 231-BR cells support the hypothesis that cancer cells acquire their expression to adapt to the brain microenvironment. We confirmed specific upregulation of LYPD1, TMEM47, and SLITRK2 in 231-BR cells compared with other cell types, as these genes are not part of any previously described brain metastatic signatures (Figure 1A). ESM1, which is upregulated in all metastatic cell types compared with parental or TMD-231 cells in the microarray assay, also showed higher expression levels in LMD, BMD, ADMD-231 cells and 231-BR cells compared with parental or TMD-231 cells by qRT-PCR (Figure 1A). Results of technical replicates are shown because of wide variation in fold induction between experiments. For example, the level of TMEM47 was higher in 231-BR by 193-, 92-, 153-, 891- and 631-fold compared with MD-231P cells in five experiments. Similarly, LYPD1 levels were higher by 19-, 49-, 6-, 49- and 44-fold in 231-BR cells compared with MD-231P cells in five experiments (see also Figure 5 below for statistical analysis).

**Table 1:** Genes overexpressed (>2 fold, *p* < 0.0002) in 231-BR compared with cells metastasized to other organs Genes shown in bold have neuronal functions.

Genes	*p* value	Fold change 231-BR vs. other metastasis
**TMEM47**	6.83E-11	8.92
**LYPD1**	9.89E-12	6.18
CD96	8.2E-06	5.89
**TFAP2C**	3.65E-06	5.61
**SLITRK2**	4.37E-08	5.58
LOC10013407	2.58E-10	3.56
**EEF1A2**	4.40E-05	3.49
SHISA2	9.84E-06	3.48
AKR1C3	9.67E-05	3.23
MYH10	2.36E-05	2.99
**HOXB5**	3.06E-06	2.98
**NINJ2**	3.70E-05	2.95
Hs.580229	1.52E-08	2.82
**SERPINF1**	3.07E-07	2.80
**CPE**	8.68E-05	2.76
**MAGEE1**	4.11E-06	2.72
GZMA	0.000157	2.53
**RPPH1**	8.27E-06	2.51
C17ORF70	5.68E-06	2.50
C8ORF13	0.00013	2.31
ZNF204	0.000183	2.28
SPIN4	2.28E-05	2.27
CLGN	9.05E-07	2.16
NCKAP1L	1.86E-09	2.14
TIE1	8.02E-06	2.08

LYPD1, TMEM47, and SLITRK2 are transmembrane proteins likely involved in ligand-dependent signal transduction. SLITRK2 family genes, including SLITRK2, are expressed predominantly in brain [28]. These genes likely play critical roles in cancer progression because cBioPortal analysis revealed amplification and /or mutations of TMEM47, LYPD1 and SLITRK2 in a variety of cancers including breast cancer [30]. Four percent of patient-derived breast cancer xenografts in the cBioPortal show amplification of LYPD1 and mutation in SLITRK2. However, there are limited reports on the function of these proteins. To gain insight into their function, we analyzed the STRING database to identify potential interacting partners [31]. While no proteins interacting with TMEM47 were found, LYPD1 and SLITRK2 appear to be involved in various signaling including cell adhesion and neurotransmitter signaling (Figure 1B). For example, GPR39, a G-protein-coupled receptor expressed mostly in brain, is at the top of the list of LYPD1 interacting partners [32]. Neuronal pentraxin-1 is the major interacting partner of SLITRK2, which mediates synaptic remodeling [33]. Future studies will determine the critical role played by these proteins in adaptation of brain metastatic cells to the brain microenvironment.

We created another brain metastatic variant from BMD-231 cells. A nude mice injected with BMD-231 cells via intra-cardiac route developed brain metastasis and metastatic cells were established in culture. These cells, called 231-BR-2, overexpressed TMEM47 but not other genes tested compared with BMD-231 cells (Figure 1C). We also observed elevated TMEM47 expression in brain metastatic variant of MCF-7 cells overexpressing HER2 (MCF-7HER2-BR) compared with parental MCF-7 cells overexpressing HER2 oncogene (Figure 1D) [34]. Thus, TMEM47 is a new brain metastasis-associated gene. Please note that CT values of SLITRK2 expression in 231-BR-2 and MCF-7HER2-BR cells were above 30 and thus are not reliable.

**Figure 2:**
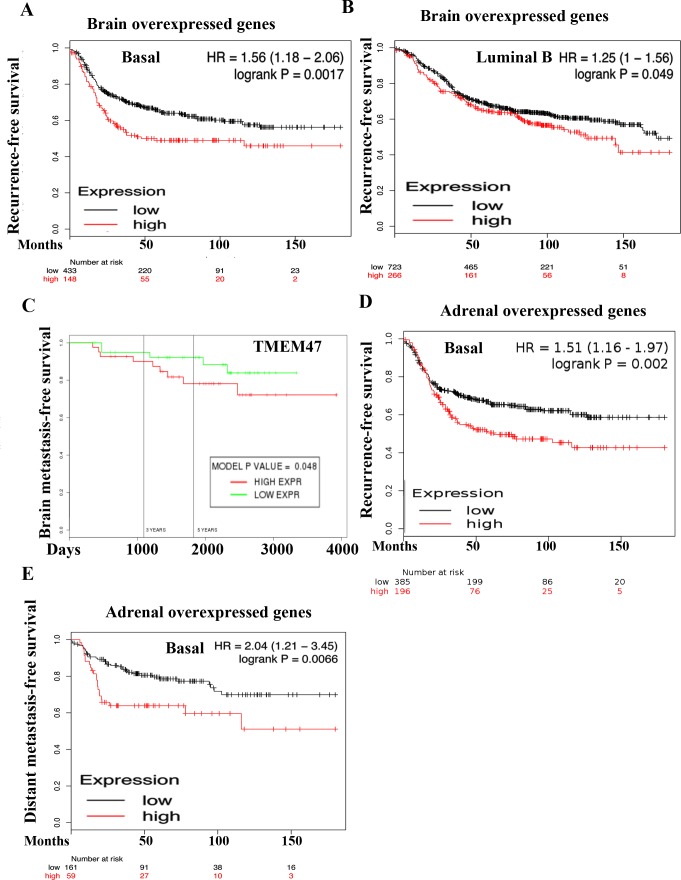
Prognostic value of genes overexpressed in 231-BR and ADMD-231 cells **A**) Elevated expression of 231-BR overexpressed genes (TMEM47, LYPD1, CD96, TFAP2C, EEF1A2, DDX, MYH10, HOXB5, NINJ2, SERPINF1, CPE, MAGEC2, CTLA3, C17orf70, ZNF704, NCKAP1L, and TIE1) in primary breast tumor is associated with poor recurrence-free survival among patients with basal breast cancer. Patients were split by median to classify into high or low expressers. **B**) Elevated expression of 231-BR specific genes in luminal B breast cancer is also associated with poor recurrence-free survival. **C**) TMEM47 overexpression is associated with poor brain metastasis-free survival. **D**) Elevated expression of ADMD-231 overexpressed genes (CYB5R2, TAGLN, HAND1, RAB3IL1, TRMT12, TSPAN8, MMP3, STXBP6, AP1S2, and HSPB8) in primary tumor is associated poor recurrence-free survival among patients with basal breast cancer. **E**) ADMD-231 overexpressed genes are also associated with poor distant metastasis-free survival among patients with basal breast cancer.

Analysis of a public database [35], which contains gene expression data in on primary tumors but not metastases, for the prognostic value of combined expression of the top 17 genes overexpressed (> 2 fold, p<0.0001, TMEM47, LYPD1, CD96, TFAP2C, EEF1A2, DDX, MYH10, HOXB5, NINJ2, SERPINF1, CPE, MAGEC2, CTLA3, C17orf70, ZNF704, NCKAP1L and TIE1) in 231-BR cells for which data were available showed elevated expression correlating with poor recurrence-free survival of patients with basal or luminal B breast cancer (Figure 2A and 2B). With respect to brain metastasis-free survival, overexpression of TMEM47 was associated with poor brain metastasis-free survival [20] (Figure 2C). TMEM47 displayed prognostic value in estrogen receptor and progesterone negative but not in estrogen receptor and progesterone receptor positive breast cancer (data not shown). LYPD1 and SLITRK2 did not show any significance.

We next determined whether the expression of LYPD1, TMEM47 and/or SLITRK2 is enriched in a specific intrinsic subtype of breast cancer. Three public datasets were analyzed (GSE2607, GSE10886, and GSE19783) [36-38]. LYPD1 but not SLITRK2 showed a trend of elevated expression in basal subtype but differences reached statistical significance only in the GSE19783 dataset (Figure S1 and Figure S2). In this dataset, TMEM47 also showed elevated expression in Basal and HER2+ breast cancers compared with luminal A and B breast cancers. Thus, overexpression of these genes may not be unique to a subtype of breast cancer. Alternatively, the brain microenvironment influences the expression of these genes in metastatic cells irrespective of the subtype.

Since no adrenal metastasis signature has been defined so far, we examined 10 genes overexpressed in ADMD-231 (>1.5 fold, *p* < 0.01) compared with other metastases for prognostic relevance. Genes were selected based on the availability of data in the public database and included CYB5R2, TAGLN, HAND1, RAB3IL1, TRMT12, TSPAN8, MMP3, STXBP6, AP1S2, and HSPB8 [35]. Overexpression of these genes was associated with poor recurrence-free survival and distant metastasis-free in basal breast cancer (Figure 2D and 2E). Please note that these genes did not show prognostic relevance in other intrinsic subtypes of breast cancer.

### Genes differentially expressed in organ-specific metastatic cells were linked to unique and shared signaling networks

**Figure 3:**
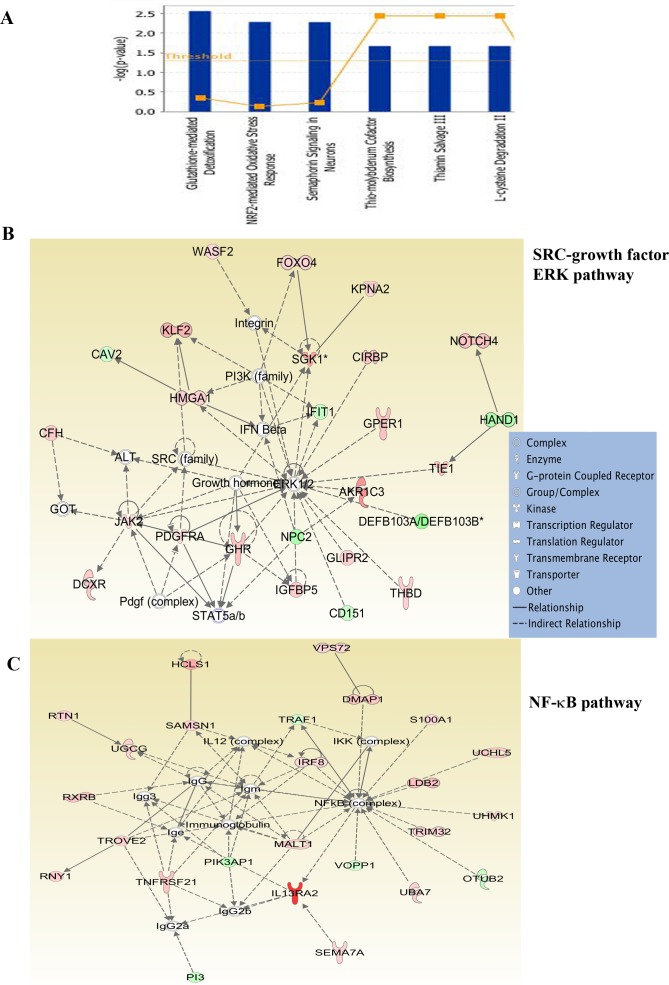
Ingenuity pathway analysis of genes differentially expressed in 231-BR cells **A**) Major signaling pathways in 231-BR cells. **B**) 231-BR cells show activation of SRC-ERK-growth hormone network. **C**) NF-κB signaling network is active in 231-BR cells. Genes labeled in red are overexpressed, whereas genes in green are expressed at lower levels in 231-BR cells compared with other metastatic cells.

**Figure 4:**
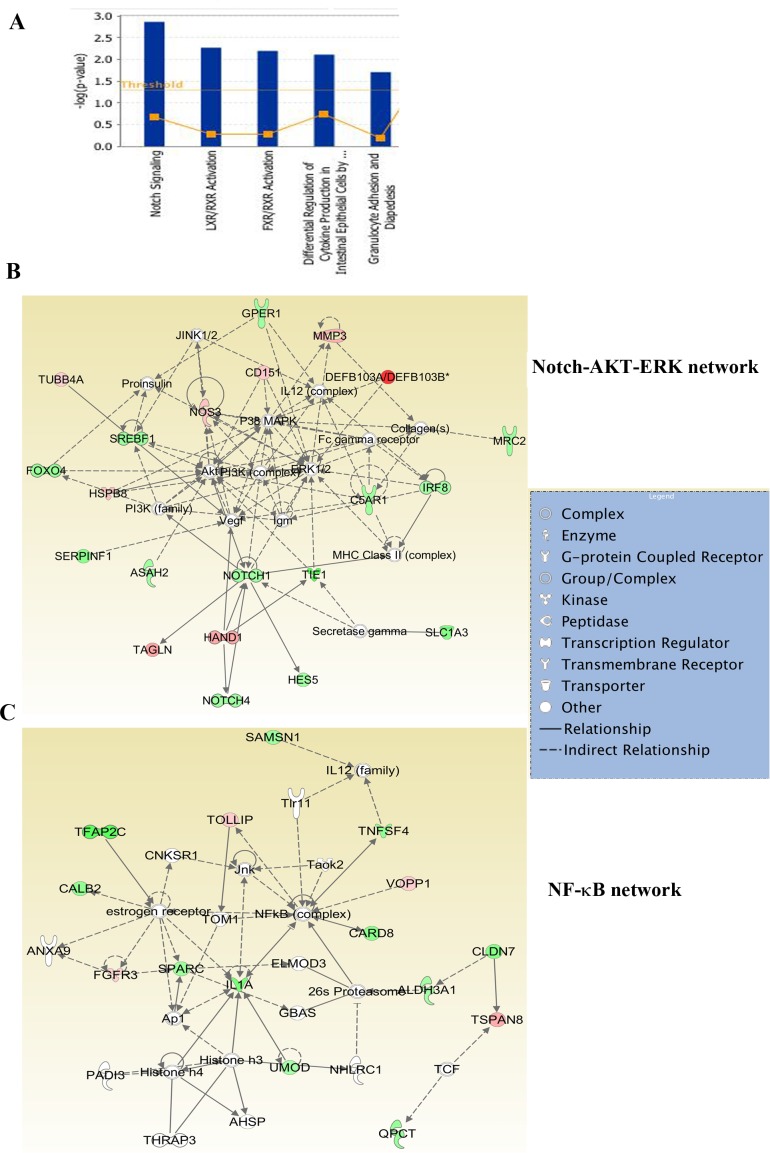
Ingenuity pathway analysis of genes differentially expressed in ADMD-231 cells **A**) Notch, FXR/RXR and LXR/RXR networks involved in steroidogenesis similar to adrenal gland are the major pathways in ADMD-231 cells. **B**) ADMD-231 cells show activation of Notch-ERK-AKT network. **C**) NF-κB signaling network is active in ADMD-231 cells.

To determine the signaling pathways active in cells isolated from different sites of metastasis, we subjected differentially expressed genes to Ingenuity pathway analysis. Glutathione-mediated detoxification, NRF2-mediated oxidative stress response, and Semaphorin signaling in neurons are a few of the signaling pathways in 231-BR cells (Figure 3A). The top two networks in 231-BR cells included SRC-ERK-growth hormone and NF-κB (Figure 3B and 3C). SRC pathway activation has also been noted previously in the BT474 HER2-positive cell brain metastasis model [18]. Notch, LXR/RXR and FXR/RXR pathways are the three major pathways activated in ADMD-231 cells (Figure 4A). Networks in these cells included Notch-ERK-AKT and NF-κB (Figure 4B and 4C).

LMD-231 cells showed activation of acute phase response signaling, primary immunodeficiency signaling, and glutamate receptor signaling (Figure S3A). Networks included TNF-CEBPA-p53 and NF-κB (Figure S3B and S3C). Involvement of CEBPA in the network is interesting because of its critical role in lung maturation [39]. BMD-231 cells displayed activation of cytokine signaling, hematopoiesis from pluripotent stem cells and JAK1/JAK3 cytokine signaling (Figure S4A). Signaling networks in these cells included ERK-growth hormone and TNF-p53 (Figure S4B and S4C). Activation of neuronal, orphan nuclear receptor, acute phase response, and cytokine signaling in brain, adrenal, lung, and bone metastatic cells, respectively, further suggests organ-specific adaptive response in metastatic cells.

### 231-BR cells displayed elevated NF-κB DNA binding activity, which was sensitive to DMAPT

**Figure 5:**
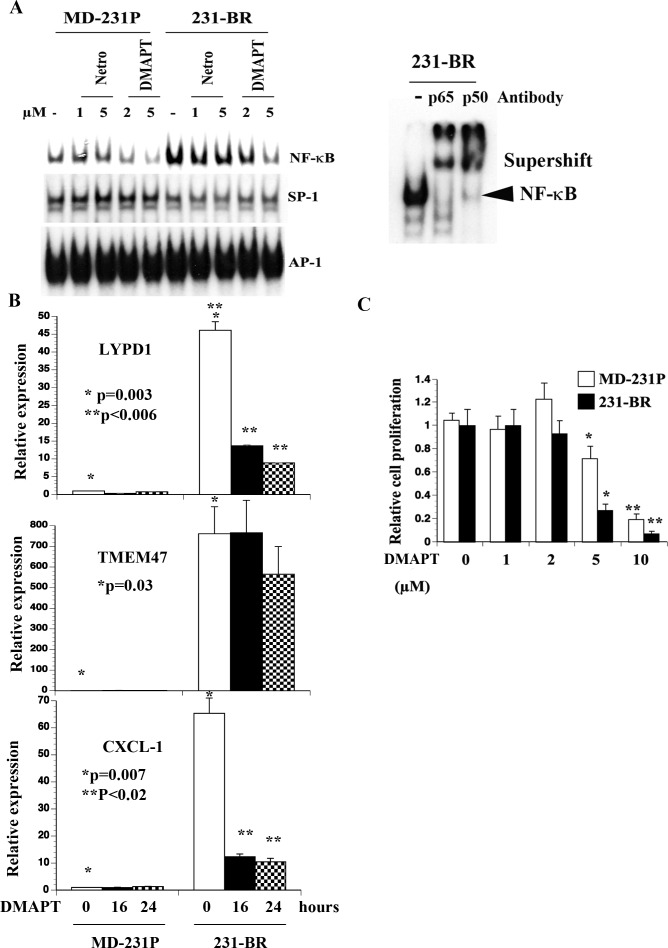
Elevated NF-κB activity in 231-BR cells compared with parental cells **A**) DMAPT but not netropsin (netro) inhibited NF-κB DNA binding activity in 231-BR cells. Supershift assays showed p50:p65 NF-κB complex in 231-BR cells. **B**) DMAPT (10 μM) reduced CXCL1 and LYPD1 but not TMEM47 expression. qRT-PCR was performed to measure mRNA levels. * *P* values MD-231P versus 231-BR; ** *P* values untreated 231-BR versus DMAPT-treated 231-BR cells. **C**) DMAPT inhibited proliferation of 231-BR cells.

To extend the above observation from Ingenuity pathway analysis, we examined NF-κB DNA binding activity in MD-231P and 231-BR cells by electrophoretic mobility shift assays (EMSAs). As we reported previously [40], MD-231P cells displayed constitutive NF-κB DNA binding activity, which was further elevated in 231-BR cells (Figure 5A). NF-κB:DNA complex contained p65 and p50 subunits as per super-shift assay. We next examined the effects of netropsin, which inhibits NF-κB when DNA binding is dependent on HMGA2 [41], and DMAPT, a direct NF-κB inhibitor. DMAPT is a water-soluble parthenolide derivative and has been characterized for anti-tumor activity *in vitro* and *in vivo* [42-45]. While netropsin had minimum effect, DMAPT significantly reduced NF-κB DNA binding activity (Figure 5A). 231-BR cells expressed ~65-fold higher levels of CXCL1, an NF-κB inducible chemokine involved in metastasis [46], compared with MD-231P cells, which was reduced by DMAPT (Figure 5B). DMAPT reduced the expression levels of LYPD1 (from 50 –fold to 10 fold) but not TMEM47 suggesting that NF-κB controls the expression of select genes of the brain metastasis signature (Figure 5B). In cell proliferation assays, while both MD-231P and 231-BR cells were sensitive to DMAPT, the concentration of drug required to inhibit 231-BR cells was lower than that for MD-231P cells (*p* = 0.0001) suggesting that 231-BR cells are dependent on NF-κB for survival (Figure 5C).

### 4T1-BR cells showed elevated NF-κB compared with 4T1 cells and were sensitive to DMAPT

**Figure 6:**
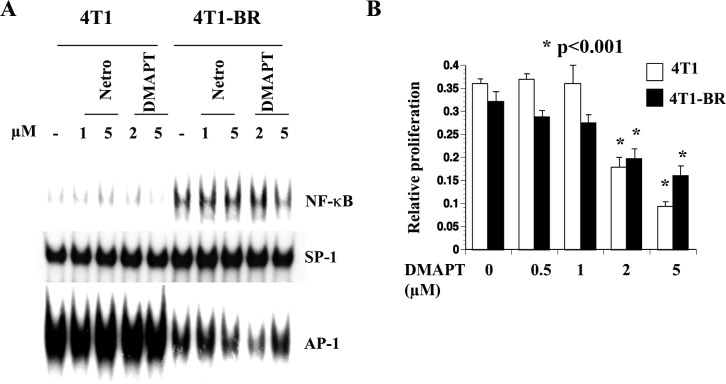
4T1-BR cells displayed elevated NF-κB activity compared with 4T1 cells **A**) DMAPT inhibited NF-κB activity in 4T1-BR cells. Note lower AP-1 DNA binding activity in 4T1-BR cells compared with 4T1 cells. **B**) DMAPT inhibited 4T1-BR cell proliferation.

To determine whether elevated NF-κB activity is observed in additional brain metastasis models, we compared NF-κB in parental 4T1 and a brain-seeking variant of this cell line [45]. 4T1 cells are derived from a spontaneous mammary tumor in BALB/c mice and form highly metastatic tumors upon mammary fat pad injection in syngeneic mice [47]. NF-κB DNA binding activity was elevated in 4T1-BR cells compared with parental 4T1 cells and DMAPT reduced this binding activity (Figure 6A). Note that AP-1 DNA binding activity was lower in 4T1-BR cells compared with 4T1 cells suggesting transcription factor switch with specific upregulation of NF-κB in brain metastatic cells. Unlike 231-BR cells, 4T1-BR cells and parental 4T1 cells were similarly sensitive to DMAPT (Figure 6B). Thus, brain metastatic cells in both model systems show elevated NF-κB activity and can potentially be targeted by NF-κB inhibitors.

## DISCUSSION

There have been several attempts to identify genes associated with brain metastasis and to functionally validate these genes for imparting blood brain barrier extravasation, vascular co-adaptation, interaction with brain microenvironment, and cell survival function. Using brain metastatic cell lines derived from four different models, Valiente et al. showed upregulation of seven genes in three out of four models [16]. The authors then focused on SERPIN1 and SERPINB2 and demonstrated their role in establishing vascular adaptation in brain [16]. Although SERPIN1 was not one of the upregulated genes in 231-BR cells (1.22-fold increase but p=0.18), significant upregulation of SERPINB2 and SERPINIF1, a serpine family member without protease inhibitory activity but with neurotropic activity [48], was observed in 213-BR cells compared with other metastatic cells (Table S2). Among the other six genes (CTCF, DUSP1, GALC, HIST1HIC, LEF1, and PCDH7), we found upregulation of DUSP1 and GALC in all metastatic cells compared with parental cells, irrespective of sites of metastasis. Among the recently described DNA repair genes upregulated in brain metastatic cells [49], we noted upregulation of RAD51 (1.1-fold, *p* = 0.03) and RAD51C (1.4 fold, *p* = 0.016) but not BARD1 in 231-BR cells compared with other metastatic cells (Table S2). However, 231-BR cells did not show specific changes in the expression levels of the recently described BRCA1 deficient-like gene signature enriched in the brain metastasis of HER2+ breast cancer patients [50, 51]. Nonetheless, four among 13 genes of this signature (NDRG1, CCND1, BOP1, and Myc) were upregulated in metastatic cells irrespective of sites of metastasis compared with parental or TMD-231 cells (Table S2). Similarly, we did not find any overlap between the brain metastasis signatures described in our study and the signature described by Salhia *et al*. [52]. However, the CRYAB gene, which was downregulated in the brain metastasis in the study described by Salhia et al was also downregulated in all metastatic cells in our analysis. Differences in the types of comparison adapted in different studies may partly be responsible for minimum overlap in genes between signatures. For example, our evaluation involved comparison between parental and metastasis as well as between organ-specific metastatic cells whereas other studies compared brain metastasis with only primary tumor.

Glutathione-mediated detoxification, NRF2-mediated oxidative response, and Semaphorin signaling pathways activated in brain metastatic cells suggest a unique biology of these metastatic cancer cells and potentially explain their relative resistance to standard chemotherapy and possibly challenges the widely held belief that poor BBB permeability of chemotherapeutic drugs is the main reason for treatment failure. Inherent ability to detoxify these drugs may be one of the main reasons for treatment failure. In this respect, a recent study has shown that physical interaction between cancer cells and astrocytes leads to upregulation of glutathione transferase 5A, which contributes to drug resistance [53]. DMAPT, the NF-κB inhibitor tested in this study, has previously been shown to deplete glutathione and cause the death of leukemic cells [54]. Thus, sensitivity of 231-BR cells to DMAPT could be related to their dependency on glutathione and the NF-κB signaling network and the ability of DMAPT to inhibit both pathways. We also noted activation of a signaling network involving SRC kinases in 231-BR cells (Figure 3B), which has recently been suggested to be a therapeutic target for brain metastasis [18].

Although there have been large efforts in defining gene expression signatures for bone and lung metastases [55-57], an adrenal gland metastasis signature is yet to be described. Minn et al. described MDA-MB-231 variants that metastasize to both lungs and adrenal or bone and adrenal but did not define an adrenal-specific gene expression signature [58]. This study, to our knowledge, describes the first adrenal metastatic signature for breast cancer. Ten genes, which are expressed 1.5-fold (p<0.01) higher in ADMD-231 compared with other metastatic cells, displayed prognostic significance in basal breast cancer but not in other subtypes (Figure 2). There is limited literature on the consequences of adrenal metastasis, although a rare case of adrenal failure due to adrenal metastasis in breast cancer has been reported [59]. Transcriptome changes associated with adrenal metastasis were significantly higher compared with bone or lung metastasis (*p* = 0.0001, Chi-square with Yates correction) suggesting the need for substantial genomic changes to achieve this metastasis. Notch and orphan nuclear receptor signaling were dominant in adrenal metastatic cells. The Notch pathway plays a major role in adrenal gland development, whereas LXR signaling controls steroidogenic pathways in the adrenal gland [60]. Thus, it is likely that cancer cells that have metastasized to the adrenal gland undergo genomic changes that help them to adapt to the adrenal gland or primary tumor cells with these pathways activation metastasize preferentially to the adrenal gland.

The majority of studies on breast cancer metastasis utilized MDA-MB-231 cells. This cell line represents the claudin-low/mesenchymal subtype, which overexpresses stem cell-enriched genes [61] and has a natural tendency to metastasize to brain and lungs. Brain and lung metastatic signatures developed using this cell line have shown clinical utility [62]. Several of the genes that were part of the original bone metastatic signature developed using this cell line, including CXCR4, CTGF, and MMP1, were part of the general metastatic signature irrespective of organ-specificity (Table S1) [55]. Because prior knowledge exists on a lung metastatic signature, we did not perform an extensive analysis. However, identification of acute phase response and glutamate signaling networks in lung metastatic cells suggests an adaptive response of these cells to lung.

In summation, our results indicate that organ-specific metastatic cells acquire the ability to adapt to sites of metastasis, which may involve genomic or epigenomic changes. We observed general upregulation of transcription in metastatic cells compared with parental cells suggesting that genomic aberrations leading to enhanced RNA polymerase II activity are sufficient for metastasis. Alternatively, genomic/epigenomic changes in a few primary tumor cells may activate organ-specific adaptive gene networks prior to their exit from the primary site. In this respect, patients with primary tumors that overexpressed brain or adrenal metastasis signature genes had poor outcome (Figure 2). While inhibitors of NF-κB signaling can be potential chemosensitizers of the majority of metastases as we have demonstrated previously with lung metastasis [44], organ-specific signaling networks identified in this study can be used to develop drugs targeting specific sites of metastasis to limit toxicity. In addition, our study identified TMEM47, a transmembrane protein with little known biology, as a brain metastasis associated gene. TMEM47 could potentially be developed as a biomarker and targeted therapeutically based on its cellular localization at the membrane.

## MATERIALS AND METHODS

### Cell lines

MDA-MB-231 and its mammary fat pad tumor and metastatic derivatives have been described [23]. 231-BR and its parental counter part MD-231P, 4T1 and 4T1-BR cells have been described [49, 63]. We also generated a new brain metastatic variant (231-BR-2) from a nude mouse that developed brain metastasis after intra-cardiac injection of bone metastatic variant (BMD-231). Despite the potential for genomic drift during growth within animals and/or during culturing after isolation from sites of metastasis, cell line identification using short tandem repeat profiling (DNA Diagnosis Center, Fairfield, Ohio, USA and Genetica DNA Laboratories, Cincinnati, Ohio, USA) confirmed that all variants are genomically similar to parental MDA-MB-231 cells. MDA-MB-231 and its derivatives were maintained in MEM plus 10% FBS, whereas 4T1 and 4T1-BR cells were maintained in DMEM plus 10% FBS.

### RNA preparation, microarray and quantitative reverse transcription polymerase chain reaction (qRT-PCR)

RNA was prepared using RNeasy kit (Qiagen, Valencia, CA, USA) and cDNA from four μgs of RNA was synthesized using the cDNA Synthesis kit (Bio-Rad, Hercules, CA, USA). qRT-PCR was performed using SyberGreen on a TaqMan 7900HT instrument (Applied Biosystems, Carlsbad, CA, USA). Sequences of primers used for qRT-PCR are listed in the supplementary Table S3. Illumina HumanHT-12 V4 expression beadchip was used for microarray analysis of biological triplicates. Genes that showed insignificant signals in a majority of samples were removed and only those probes that showed statistically significant signal in at least half of samples of at least one group were retained. The probe level data were then collapsed to gene level data by retaining only the probes, which showed a maximal coefficient of variation across all samples. The data were imported into Partek genomics suite for differential expression analysis. ANOVA analysis was performed to identify genes differentially expressed between different cell lines. Genes differentially expressed at *p* value of <0.01 were considered further for pathway analysis using Ingenuity pathway analysis software (© Ingenuity systems, CA, USA). Prognostic relevance of overexpressed genes was determined using a public database as well as a new database created by us [35, 64]. Microarray dataset is available in the Gene Expression Omnibus (GSE66495).

### Generation of metastasis signatures

Predictive analysis of Microarray (PAM) [25] was used to identify signature genes capable of discriminating phenotypes with minimal classification error. PAM analysis was performed on a 1 × Rest basis comparing one phenotype with all other phenotypes to identify a group of genes that could differentiate that phenotype from the rest of phenotypes arriving at gene signatures specific for each phenotypic group. Thresholds for PAM analysis were adjusted to identify the smallest possible gene signature with minimal misclassification error rate.

### Electrophoretic Mobility Shift Assays (EMSA) and cell proliferation studies

EMSA with whole cell lysates was performed as described previously [65]. Probes for the assay were purchased from Promega (Madison, WI, USA), whereas antibodies for supershift were from EMD Millipore (Billerica, MA, USA). For cell proliferation studies, 1000 cells/well were plated on a 96-well plate and cells were treated with DMAPT (eight wells per drug and highest and lowest numbers were discarded during analysis). Cell proliferation was measured 48 hours after drug treatment using Bromodeoxyuridine-ELISA (Calbiochem/EMD Millipore). Results are from two or more independent experiments.

### Statistical analysis

Graphpad Prism (Graphpad.com) was used for statistical analysis of qRT-PCR and cell proliferation assay. Analysis of variance was used to determine the *P*-values between mean measurements. A *P*-value of <0.05 was deemed significant.

## SUPPLEMENTARY MATERIAL, FIGURES AND TABLES






