# Supplementary material for: Organ-specific adaptive signaling pathway activation in metastatic breast cancer cells
Source: Oncotarget. 2015 Mar 30;6(14):12682–96. doi: 10.18632/oncotarget.3707 (PMC4494966; doi:10.18632/oncotarget.3707)
Supplement: Supplementary file 1 [file oncotarget-06-12682-s001.pdf]

Organ-specific adaptive signaling pathway activation in metastatic breast cancer cells

Supplementary Material

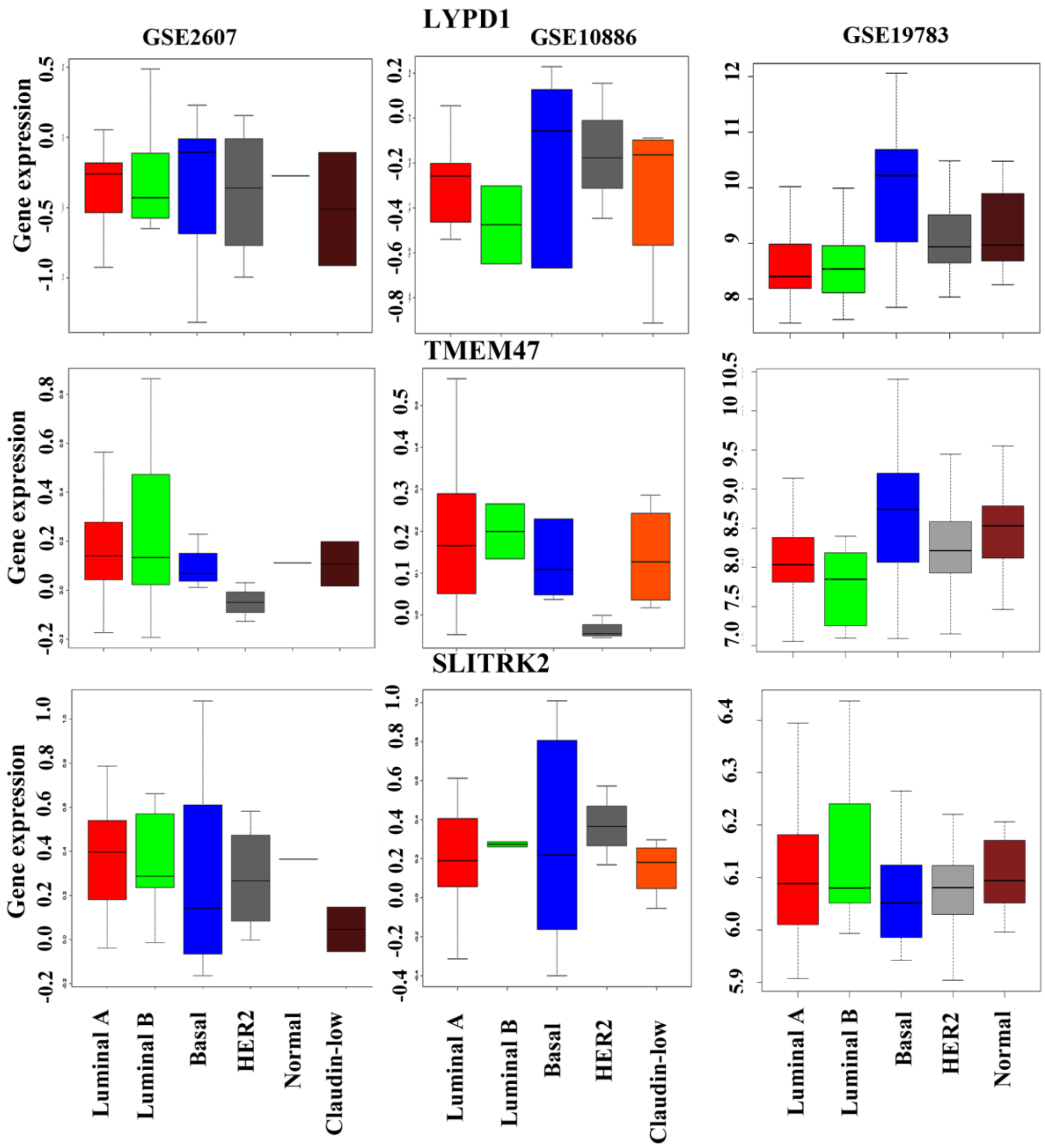

Figure S1: Expression levels of LYPD1, TMEM47, and SLITRK2 in various subtypes of breast cancer. Datasets used for the analyses are indicated. Normalized data were used for these analyses.

			GSE 2607		GSE 10886		GSE 19783	
			PVAL	RATIO	PVAL	RATIO	PVAL	RATIO
TMEM47	LUMINAL A	LUMINAL B	0.5353	0.597437326	0.9189	0.952836637	0.0167	0.509908991
	LUMINAL A	BASAL	0.6043	1.38018018	0.5898	0.606673114	0.1478	-0.419591212
	LUMINAL A	HER2	NA	-3.126530612	0.0018	-5.702727273	0.7107	0.079641536
	LUMINAL A	NORMAL	0.0189	1.367857143	NaN	NaN	0.3228	-0.26177778
	LUMINAL A	CLAUDIN	0.7233	1.418518519	0.5513	1.367560497	NaN	NaN
	LUMINAL B	BASAL	0.3684	2.31016731	0.6229	0.636702128	0.0014	-0.929500203
	LUMINAL B	HER2	0.0816	-5.233236152	0.157	-5.985	0.0185	-0.430267455
	LUMINAL B	NORMAL	NA	2.289540816	NaN	NaN	0.0031	-0.771686771
	LUMINAL B	CLAUDIN	0.4193	2.374338624	0.5545	1.435251799	NaN	NaN
	BASAL	HER2	0.0141	-2.265306122	0.1587	-9.4	0.0667	0.499232748
	BASAL	NORMAL	NA	0.991071429	NaN	NaN	0.6029	0.157813432
	BASAL	CLAUDIN	0.9797	1.027777778	0.4559	2.254196643	NaN	NaN
	HER2	NORMAL	NA	-0.4375	NaN	NaN	0.161	-0.341419316
	HER2	CLAUDIN	0.3114	-0.453703704	0.0672	-0.239808153	NaN	NaN
	NORMAL	CLAUDIN	NA	1.037037037	NaN	NaN	NaN	NaN
LYPD1	LUMINAL A	LUMINAL B	0.9953	1.004270463	0.781	0.852690947	0.9309	0.016257026
	LUMINAL A	BASAL	0.8049	0.837388724	0.8471	0.81553043	3.00E-04	-1.369349923
	LUMINAL A	HER2	0.7127	0.724053881	0.3244	2.593525877	0.0103	-0.529117743
	LUMINAL A	NORMAL	NA	1.029927007	NaN	NaN	0.0171	-0.590920017
	LUMINAL A	CLAUDIN	0.6722	0.553333333	0.7757	1.220329708	NaN	NaN
	LUMINAL B	BASAL	0.82	0.833827893	0.9643	0.956419712	4.00E-04	-1.385606949
	LUMINAL B	HER2	0.7247	0.720974984	0.2948	3.041577825	0.0229	-0.54537477
	LUMINAL B	NORMAL	NA	1.025547445	NaN	NaN	0.0259	-0.607177043
	LUMINAL B	CLAUDIN	0.6717	0.550980392	0.6183	1.431151242	NaN	NaN
	BASAL	HER2	0.8688	0.864656831	0.4894	3.180170576	0.0194	0.84023218
	BASAL	NORMAL	NA	1.229927007	NaN	NaN	0.0374	0.778429906
	BASAL	CLAUDIN	0.7456	0.660784314	0.738	1.49636318	NaN	NaN
	HER2	NORMAL	NA	1.422445255	NaN	NaN	0.8151	-0.061802273
	HER2	CLAUDIN	0.8252	0.764215686	0.5319	0.47052922	NaN	NaN
	NORMAL	CLAUDIN	NA	0.537254902	NaN	NaN	NaN	NaN
SLITRK2	LUMINAL A	LUMINAL B	0.9298	1.029799764	0.4987	0.778367618	0.9129	-0.006052785
	LUMINAL A	BASAL	0.652	1.307283035	0.7963	0.75718128	0.302	0.054871901
	LUMINAL A	HER2	0.5422	1.345421903	0.3365	0.578497452	0.884	0.010133192
	LUMINAL A	NORMAL	NA	1.026575342	NaN	NaN	0.7313	-0.022815678
	LUMINAL A	CLAUDIN	0.1023	8.058064516	0.5926	1.417094533	NaN	NaN
	LUMINAL B	BASAL	0.701	1.269453617	0.9757	0.972781065	0.2116	0.060924686
	LUMINAL B	HER2	0.6037	1.306488843	0.5019	0.743218807	0.8059	0.016185977
	LUMINAL B	NORMAL	NA	0.996868885	NaN	NaN	0.7892	-0.016762893
	LUMINAL B	CLAUDIN	0.1038	7.824884793	0.1977	1.820598007	NaN	NaN
	BASAL	HER2	0.9704	1.029174147	0.7535	0.764014467	0.4854	-0.044738709
	BASAL	NORMAL	NA	0.785273973	NaN	NaN	0.2084	-0.077687579
	BASAL	CLAUDIN	0.27	6.163978495	0.6196	1.871539313	NaN	NaN
	HER2	NORMAL	NA	0.763013699	NaN	NaN	0.662	-0.03294887
	HER2	CLAUDIN	0.2322	5.989247312	0.198	2.449612403	NaN	NaN
	NORMAL	CLAUDIN	NA	7.849462366	NaN	NaN	NaN	NaN

Figure S2: Statistical comparisons between different subtypes for the expression of LYPD1, TMEM47, and SLITRK2.

A

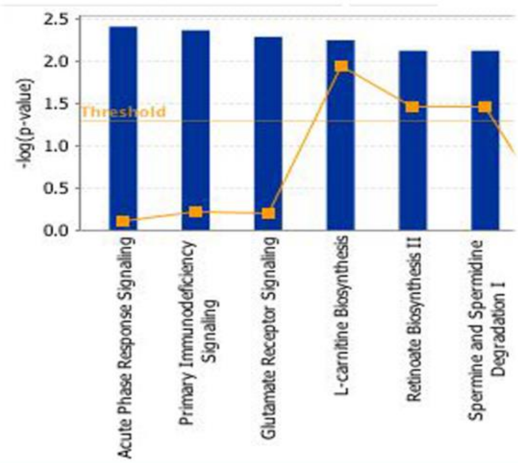

Lung metastasis genes

B

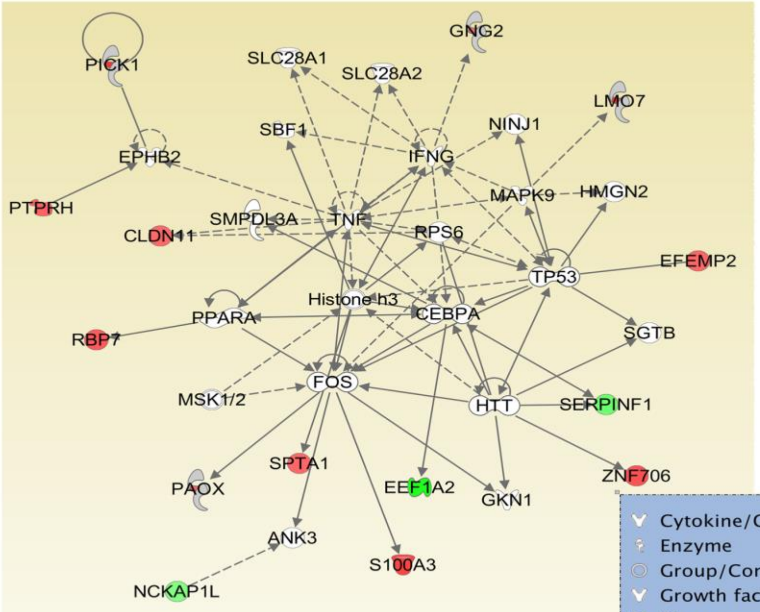

TNF-CEBPA-p53 network

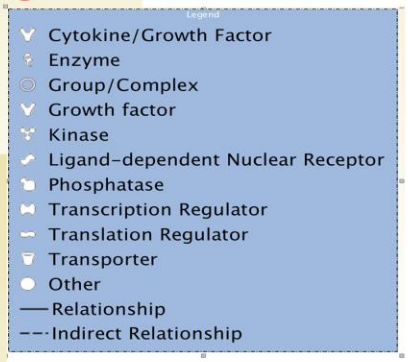

C

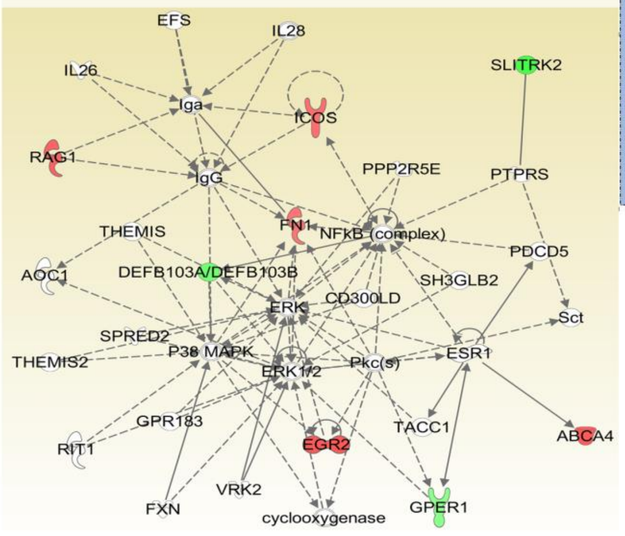

ERK-AKT-NF-κB network

Figure S3: Ingenuity pathway analysis of genes differentially expressed in LMD-231 cells. A) Major signaling pathways in LMD-231 cells. B) LMD-231 cells show activation of TNF-CEBPA-p53 network. C) ERK-AKT-NF-κB signaling network is active in LMD-231 cells.

A

Bone metastasis genes

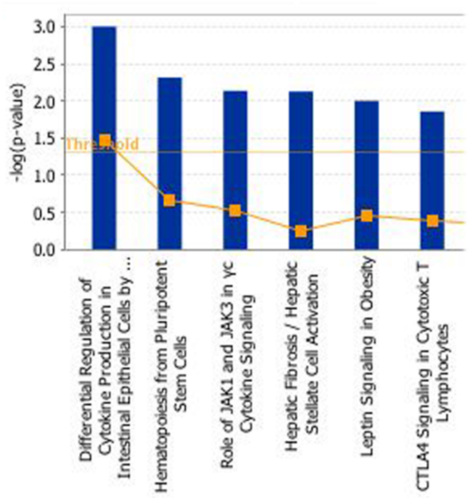

B

AKT-ERK-growth hormone network

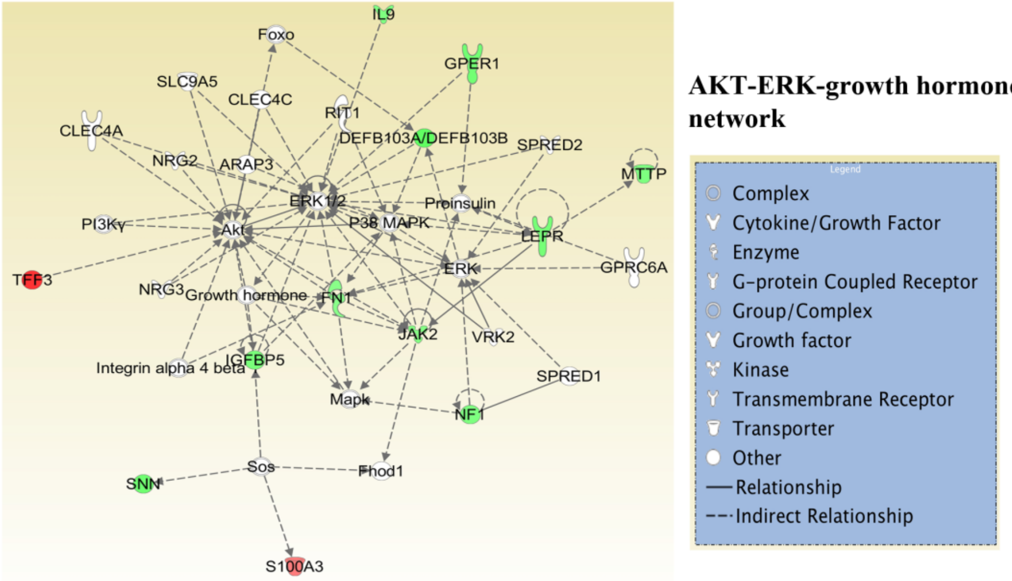

C

TNF-p53 network

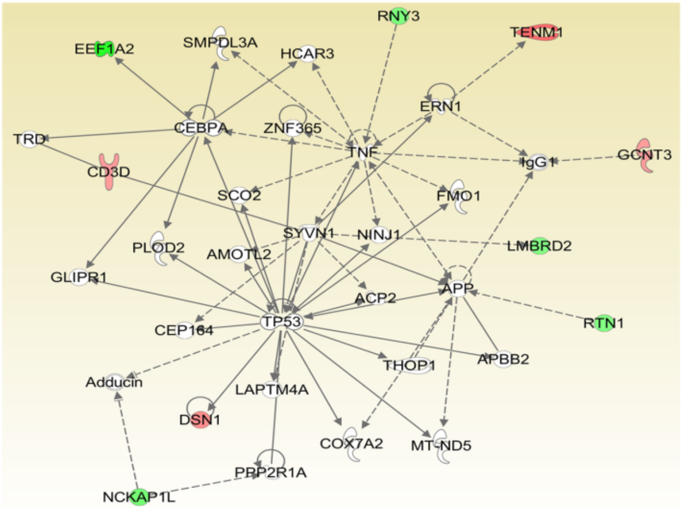

Figure S4: Ingenuity pathway analysis of genes differentially expressed in BMD-231 cells. A) Major signaling pathways in BMD-231 cells. B) BMD-231 cells show activation of AKT-ERK-Growth hormone network. C) TNF-p53 signaling network is active in BMD-231 cells.

Table S1: PAM classifier analysis of microarray results to identify organ-specific metastasis signature.

Table S2: Various comparisons of genes differentially expressed in different cell types. ANOVA analysis was performed to measure genes differentially expressed in different cell types.

Note: Tables S1 and S2 are in supplementary Information in oncotarget site.

Table S3: Sequences of primers used for qRT-PCR

Gene	Forward	Reverse
β-Actin	TGGATCAGCAAGCAG	GCATTTGCGGTGGAC
CXCL1	CCAGACCCGCCTGCTG	CCTCCTCCCTTCTGGTCAGTT
ESM1	TAATTATGCGGTGGACTGCCCTCA	TTCATGCCATCCATGCCTGAGACT
LYPD1	TTGCGGATTGTTCTTGCTTCCAGG	TGATGATGCACAGGACTTGCGGTA
SGK1	AGGCATGTAGCAGGGAGGCTTATT	TTTCATTCTTCGGGTTGCCCAAGG
SLITRK2	TCGGTTACTAATGACTTGGCGGCT	TTAGGCACCTTCGGTGTCAACTCA
TMEM47	TTACCGCCTGCTTATCCTACCCAA	TCCCATTAGATGCTGAAGGGCAGT
